# Does p53 codon 72 polymorphism have a prognostic value in carcinoma of the vulva and vagina?

**DOI:** 10.1007/s12032-017-0893-6

**Published:** 2017-01-31

**Authors:** Alvida Qvick, Bengt Sorbe, Gisela Helenius, Mats G. Karlsson, Gabriella Lillsunde Larsson

**Affiliations:** 1 Department of Laboratory Medicine, Faculty of Medicine and Health, Örebro University, Örebro, Sweden; 2 Department of Oncology, Faculty of Medicine and Health, Örebro University, Örebro, Sweden

**Keywords:** P53, Codon 72, Polymorphism, Vagina, Vulva, Carcinoma, HPV

## Abstract

Human papilloma virus (HPV) is considered to be responsible for a large part of vaginal and vulvar carcinomas, and the p53 codon 72 polymorphism has been implicated in susceptibility to cancer induced by this virus, but with contradicting results. In this study, we have investigated the prognostic value of the codon 72 polymorphism by real-time PCR (qPCR) in two cohorts of vaginal (*n* = 66) and vulvar (*n* = 123) carcinomas. In vaginal carcinoma, arginine homozygous patients were significantly associated with a higher primary cure rate (*p* = 0.023) but also associated with a higher recurrence rate (*p* = 0.073), significant at distant locations (*p* = 0.009). No significant differences were found in overall survival rate (*p* = 0.499) or cancer-specific survival rate (*p* = 0.222). A higher frequency of arginine homozygosity was noted in HPV-positive tumors (*p* = 0.190) in comparison with HPV-negative tumors. In vulvar carcinoma, the genotype homozygous for arginine was significantly associated with a larger tumor size at diagnosis in the entire cohort (*p* = 0.015) and a lower cancer-specific survival rate (*p* = 0.024) compared with heterozygous (arginine/proline) in HPV-negative tumors. Our results indicate that the relation between HPV and the p53 codon 72 polymorphism is complex and the significance and mechanisms responsible for this relationship need to be further elucidated.

## Introduction

Both cancers of the vagina and vulva are relatively rare cancers and account for approximately 2 and 4–5% of all gynecological cancers, respectively [1]. Vaginal and vulvar cancers mostly affect the elderly women, but an increasing incidence in somewhat younger women has been reported during the last decade [2]. Risk factors include smoking, early sexual debut, several sexual partners during life and human papilloma virus (HPV) infection [2]. Histologically, squamous cell carcinoma (SCC) accounts for 80–90% of the vaginal and 90% of the vulvar cancers with the remaining part mainly consisting of adenocarcinoma, melanoma and sarcoma [1]. SCC has in turn been further morphologically characterized into basaloid, keratinizing, non-keratinizing and verrucous carcinoma. The keratinizing form is a result of chronic vulvar dermatosis, is mostly unrelated of HPV infection, and generally affects older women, while the basaloid and warty forms are more common in relatively younger women and are associated with HPV infection [3–5].

HPV is a selective virus that infects squamous epithelia and mucous membranes and has been associated with anogenital cancers, such as cervical, penile, vulvar, vaginal and anal cancer as well as head and neck cancer [6]. In cervical carcinoma, the relation with HPV has been extensively studied and the virus is considered to be present in almost all cases [7]. Studies on HPV prevalence in vaginal and vulvar cancers have been sparse and the HPV positivity diverse, ranging between 43 and 70% in vaginal cancer [3, 4, 8–10] and 19–40% in vulvar cancer [3, 5, 11, 12], but with a unanimous result of HPV-16 being the most common genotype for both cancers. HPV-positive vaginal and vulvar cancers largely develop through a pathway shared with cervical cancer. The lower percentage of HPV-related cancers in vagina and vulva compared to cervix has been thought to depend on different morphologies and expression patterns of the cells [13] or age-related factors such as estrogen deficiency [8]. Other reasons for cancer development in these locals include occurrence of chronic inflammation or lichen sclerosus.

Survival has been related to stage and size of the tumor at diagnosis in both vaginal and vulvar carcinomas [8–11, 14]. HPV positivity has been shown to be a favorable prognostic marker for overall survival for vaginal cancer, but the same relationship has yet to be shown for vulvar cancer [4, 5, 10, 11].

The oncogenic effect of HPV is linked to its expression of two proteins, the E6 protein stimulating p53 degradation and telomerase activity, and the E7 protein binding to the retinoblastoma protein performing interactions with several other proteins important for cell growth. E6 and E7 proteins are therefore regarded as viral oncoproteins [6]. When the expression of these oncogenes is constitutively high, it can disturb the genomic stability leading to chromosomal changes which in turn give rise to activation of oncogenes or loss of tumor suppressor genes resulting in malignant growth [15]. In an HPV-positive tumor, the viral oncogenes are likely to promote the increased proliferation while in a tumor lacking a virus infection another pathway, such as affecting the p53 expression, is needed to drive the malignant progression [4, 5].

The tumor suppressor gene TP53 has an important role in inducing growth arrest and apoptosis and a dysregulation of this gene is therefore highly beneficial for cancer development [16]. In a normal cell, the protein mouse double minute 2 (MDM2) can act as an E3 ubiquitin ligase that can transfer ubiquitin to p53 which targets it to proteasome-mediated degradation. In case of an high-risk HPV infection, E6 binds to p53 and E6-associated protein ligase (E6AP) forming a complex that ubiquitinates p53 and causes its degradation [6]. TP53 has been extensively studied, and its variants, polymorphisms and mutations have been amassed in several databases. From this information, it is clear that p53 functionality is sensitive to a wide range of singe nucleotide alterations. Whibley et al. [17] raised the question as to whether some of the polymorphisms existing in a healthy population might also be of importance for p53 function under certain circumstances. The polymorphism in codon 72 of TP53 is the exchange from CCC, which encodes proline (pro), to CGC, which encodes for arginine (arg). Codon 72 is located in the proline-rich domain spanning from codon 64–92 [17]. The prevalence of the two alleles has been linked to a selective difference related to winter temperature with proline being more common in a warmer climate [18]. The frequency of the polymorphic alleles in Sweden is reported to be 9% for pro, 44% for heterozygous (arg/pro) and 47% for arg [19]. Individuals carrying the arg allele have been proposed to be more susceptible to cancer caused by HPV [20]. This has led to further analyses showing that the p53arg and p53pro differ in structure and these structural differences are most likely located in the N-terminal portion of the protein [21].

The polymorphism has been studied in relation to a wide range of cancers with inconclusive results [17]. For cervical cancer, the interest has been relatively extensive due to its strong relation with HPV infection [22, 23], while studies on vaginal and vulvar cancer have been sparse [24]. The main focus in the majority of the studies has been the importance of codon 72 on susceptibility to HPV-induced cancer with only some addressing the prognostic value [25]. Previous studies indicate that this common p53 polymorphism could affect tumor development and response to treatment. Therefore, we wished to determine the effect on prognosis and recurrence in vaginal and vulvar carcinomas of p53 codon 72 polymorphisms, which is to our knowledge the first study investigating this relation.

## Materials and methods

### Tumor samples

One hundred and thirty-three samples from patients with vulvar squamous cell carcinoma (VSCC) and 78 samples from patients with primary vaginal carcinoma were obtained from the Biobank at the Örebro University Hospital. The VSCC samples were originally collected from Örebro University Hospital, Umeå University Hospital, Uppsala University Hospital, and Central Hospitals in Eskilstuna, Falun, Gävle, Karlstad and Västerås (1983–2008). The vaginal carcinomas were originally collected from Örebro University Hospital, and from Central Hospitals in Eskilstuna, Karlstad and Västerås (1975–2002).

HPV genotyping and HPV-16 classification have been performed on both cohorts by the research team in an earlier phase [10, 11]. In these studies, three samples in the vulvar cohort with questionable HPV status were excluded and in the vaginal cohort four patients were excluded due to insufficient tumor material, four due to difficulty retrieving tumor material and one due to poor DNA quality. The samples above were excluded from this study along with seven samples in the vulvar cohort and three samples in the vaginal cohort that were excluded due to insufficient DNA material. In total, 123 cases in the vulvar cohort and 66 cases in the vaginal cohort were analyzed.

All material was retrieved from formalin-fixed and paraffin-embedded (FFPE) samples and tumors were staged at the time of diagnosis according to FIGO guidelines [26]. Histological classification was performed according to the nomenclature proposed by the World Health Organization (WHO) [27]. Clinical data were retrieved from patient records at the Department of Gynecological Oncology, Örebro University Hospital. The study was approved by the regional ethics committee board in Uppsala (Dnr 2008/294), and informed consent was given as specified in the ethical approval.

### DNA extraction

Tumor areas were marked on hematoxylin–eosin-stained sections and were punched out using a one-time disposable 1-mm skin biopsy punch (Miltex, GmbH, Germany). Tissues were deparaffinized by routine procedure, and DNA was extracted using QIAmp DNA Mini Kit (Qiagen, GmbH, Hilden, Germany) according to instructions by the manufacturer. DNA concentration was measured spectrophotometrically using a nanodrop (ND-1000, Saveen and Werner, Limhamn, Sweden).

### Real-time PCR

Samples were analyzed in 10 µl reactions with real-time PCR containing a standardized Taqman Genotyping Master Mix kit (Applied Biosystems, Foster city, CA, USA) together with 1 × Taqman SNP genotyping assays (assay ID: C_2403545_10, Applied Biosystems) and 1.8 ng/µl DNA. Initial denaturation was performed at 95 °Cfor 10 min followed by 40 cycles of 95 °C for 15 s and 60 °C for 1 min. Post-PCR allelic discrimination read was performed in 60 °C for 1 min with fluorescent markers VIC and FAM illustrating the presence of the arginine and proline allele, respectively. All runs contained a positive control for each of the three possible outcomes (arg/arg, arg/pro or pro/pro) as well as a negative control. Results were analyzed using software 7500 Fast System SDS Software (Applied Biosystems). All samples were analyzed in triplicates and in case of non-evaluable results samples were reanalyzed with the same conditions before performing a re-extraction of DNA from the tumor material.

### Statistical analysis

Survival curves were analyzed using the Kaplan–Meier method, and differences were evaluated with the log-rank test. Independent *t* test was used for comparing means and for comparison of proportions the Pearson’s Chi-square test was used. *p* values less than 0.05 were considered statistically significant. Analyses were performed using the Statistica software package (version 13, StatSoft, Inc., Tulsa, USA).

## Results

### Patient data and polymorphism

Sixty-six samples from the vaginal cohort and 123 samples from the vulvar cohort were analyzed by targeting the polymorphic variants, arginine and proline, of codon 72 in p53. In the vaginal cohort 53.0% (35 cases) were arg/arg, 37.9% (25 cases) were arg/pro, and 4.5% (3 cases) were pro/pro. In the vulvar cohort, 55.3% (68 cases) were arg/arg, 35.8% (44 cases) were arg/pro, and 4.1% (5 cases) were pro/pro (Table 1). Three samples (4.5%) from the vaginal cohort and 6 samples (4.9%) from the vulvar cohort were not classifiable and were placed in separate groups marked as “undefined.”

**Table 1:** Prevalence of the polymorphic variants of the p53 codon 72 in vaginal and vulvar carcinomas

TP53 allele	Vaginal cohort		Vulvar cohort	
No.	66	%	123	%
arg/arg	35	53.0	68	55.3
arg/pro	25	37.9	44	35.8
pro/pro	3	4.5	5	4.1
undefined	3	4.5	6	4.9

*arg* arginine, *pro* proline

The mean age of patients in the vaginal cohort with arg/arg tumors was 70.1 years (SD 12.4 years), for arg/pro genotype tumors 68.5 years (SD 11.9 years), and for pro/pro genotype tumors 65.7 years (SD 18.1 years). These differences were not significant. In vulvar carcinoma, the mean age at diagnosis of patients with arg/arg genotype tumors was 69.7 years (SD 14.5 years), for pro/arg tumors 73.8 years (SD 12.0 years), and for pro/pro genotype tumors 68.8 years (SD 11.0 years). No significant differences were found.

### HPV association and tumor characteristics

The vaginal cohort consisted of 35 HPV-positive cases and 31 HPV-negative cases. The arg/arg polymorphism (57.1%) was more common than the arg/pro variant (40.0%) in HPV-positive tumors, and the opposite was seen in HPV-negative tumors (42.9 vs. 60.0%). The difference was not statistically significant (Pearson *χ* ^2^; *p* = 0.190) (Table 2).

**Table 2:** Comprised data of HPV status, tumor stage and histology in the vaginal and vulvar carcinoma cohort compared to polymorphic variant of p53 codon 72. Data presented as number of patients with percentages shown in parenthesis

		HPV status		Tumor stage				Histology				
		Positive	Negative	I	II	III	IV	Adenocarcinoma	SCC			
Vaginal carcinoma	arg/arg	20 (57.1)	15 (42.9)	10 (28.6)	16 (45.7)	5 (14.3)	4 (11.4)	4 (11.4)	31 (88.6)			
	arg/pro	10 (40.0)	15 (60.0)	9 (36.0)	8 (32.0)	2 (8.0)	6 (24.0)	2 (8.0)	23 (92.0)			
	pro/pro	2 (66.7)	1 (33.3)	2 (66.7)	1 (33.3)	0 (0.0)	0 (0.0)	0 (0.0)	3 (100.0)			
									Basaloid	Keratinizing	Mixed	Verrucous
Vulvar carcinoma	arg/arg	20 (29.4)	48 (70.6)	17 (25.0)	26 (38.2)	15 (22.1)	10 (14.7)		9 (13.2)	45 (66.2)	14 (20.6)	0 (0.0)
	arg/pro	15 (34.1)	29 (65.9)	14 (31.8)	20 (45.5)	7 (15.9)	3 (6.8)		8 (18.2)	31 (70.5)	4 (2.3)	1 (9.1)
	pro/pro	1 (20.0)	4 (80.0)	2 (40.0)	2 (40.0)	1 (20.0)	0 (0.0)		1 (20.0)	3 (60.0)	1 (20.0)	0 (0.0)

*arg* arginine, *pro* proline, *SCC* squamous cell carcinoma

The vulvar cohort consisted of 38 HPV-positive cases and 85 HPV-negative cases. The various types of polymorphism were similarly distributed in HPV-positive and HPV-negative cases (Pearson *χ* ^2^; *p* = 0.771) (Table 2). A majority of the HPV-positive cases were positive for HPV-16 (71.4% in the vaginal cohort and 78.9% in the vulvar cohort), and the other cases were spread on different HPV types (data not shown). To achieve a reasonable number of cases in the compared groups, the HPV-16-positive cases were separated from the other types which were combined into one group. No difference in allele distributions was seen in the two constructed groups in either cohort. HPV-16 variant data were too sparse for meaningful statistical analyses in this study.

Tumor stage was not significantly associated with codon 72 polymorphism in the vaginal cohort (Table 2). In the vulvar cohort, tumors in advanced stages (III–IV) had arg/arg genotype in 25/36 (69.4%) of the cases and tumors in early stages (I–II) in 43/81 (53.1%) of evaluable cases (Pearson *χ* ^2^; *p* = 0.098) (Table 2).

Tumor size at diagnosis in the vaginal cohort was similar for tumors with arg/arg and arg/pro genotype (mean diameter 24.0 vs. 24.4 mm; *t* test, *p* = 0.896). Tumor localization in the vagina was not associated with the present genotype at codon 72 (arg/arg vs. arg/pro). However, tumors with an arg/arg genotype at codon 72 were significantly (*t* test; *p* = 0.015) larger at diagnosis (mean diameter 36 mm, SD 23 mm) than tumors with arg/pro or pro/pro genotypes (mean 25 mm, SD 14 mm) in the vulvar cohort. Data on tumor size at diagnosis were only evaluable on 84 out of 130 cases (64.6%).

Type of histology was not significantly associated with polymorphism in the vaginal cohort (Table 2). In the vulvar cohort, arg/arg genotype was more frequent (73.7%) in tumors of mixed type than in tumors of basaloid or keratinizing type (55.1%), however, not significant (Pearson *χ* ^2^; *p* = 0.133) (Table 2).

### Clinical outcome

#### Primary cure rate

##### Vaginal carcinomas

The primary cure rate of the complete series was 53 out of 66 (80.3%) cases. Among tumors achieving primary cure (complete remission) in the vaginal group, the arg/arg genotype was significantly (Pearson *χ* ^2^; *p* = 0.023) more common (66.0%) than in tumors not achieving primary cure (30.8%).

##### Vulvar carcinomas

The primary cure rate of the complete series was 116 out of 123 (88.6%) cases. The primary cure was 91.2% in the arg/arg group and 86.4% in the arg/pro genotype group (Pearson *χ* ^2^; *p* = 0.421).

#### Tumor recurrences

##### Vaginal carcinomas

Arg/arg genotype was more common (15/21, 71.4%) in tumors with recurrences (all types and sites) than in tumors with no recurrences (20/42, 47.6%) (Pearson *χ* ^2^; *p* = 0.073). In tumors with distant recurrences, this difference was more pronounced (10/11, 90.9% vs. 25/52, 48.1%) and highly statistically significant (Pearson *χ* ^2^; *p* = 0.009).

##### Vulvar carcinomas

In the vulvar cohort, the overall recurrence rate was 31/68 (45.6%) among tumors with arg/arg genotype and 17/49 (34.7%) in tumors with arg/pro or pro/pro genotype (Pearson *χ* ^2^; *p* = 0.237). Local vulvar recurrences were similar in tumors with arg/arg genotype (16/68, 23.5%) and in tumors with arg/pro or pro/pro genotype (12/49, 24.5%) (Pearson *χ* ^2^; *p* = 0.904). Inguinal lymph node recurrences were recorded in 11/68 (16.2%) tumors with arg/arg genotype and in 6/49 (12.2%) tumors with arg/pro or pro/pro genotype (Pearson *χ* ^2^; *p* = 0.552). Distant recurrences were recorded in 4/68 (5.9%) tumors with arg/arg genotype and in 4/49 (8.2%) tumors with pro/arg or pro/pro genotype (Pearson *χ* ^2^; *p* = 0.630).

#### Survival rate

##### Vaginal carcinomas

In the vaginal cohort, the arg/arg genotype was associated with a numerically, but not significantly (log-rank test; *p* = 0.609) worse 5-year cancer-specific survival (CSS) rate (43.6%) than the pro/arg genotype (53.4%). Neither overall survival rate was significantly (log-rank test; *p* = 0.554) different for the analyzed genotype groups. Patients with HPV-positive tumors and arg/arg genotype had a similar 5-year CSS (72.2%) to patients with the arg/pro genotype (80.0%) (log-rank test; *p* = 0.821). In patients with HPV-negative tumors, the rates were 53.8% for arg/arg and 43.4% for arg/pro patients (log-rank test; *p* = 0.607).

##### Vulvar carcinomas

The 5-year overall survival rate in the vulvar cohort was 40.1% for patients with tumors showing the arg/arg genotype and 67.4% for patients with tumors showing the pro/arg genotype (log-rank test; *p* = 0.065). The corresponding CSS rates were 51.5 and 72.7%, respectively (log-rank test; *p* = 0.169). When the pro/pro genotype group was combined with undefined cases and compared with the other two groups, the survival rate was very similar to the pro/arg group (73.8% 5-year cancer-specific survival rate). In the vulvar cohort, patients with HPV-positive tumors had similar CSS survival rates for the genotypes arg/arg and arg/pro (69.8 vs. 73.5%; log-rank test; *p* = 0.283). However, different CSS survival rates were observed for arg/arg and arg/pro polymorphism in HPV-negative patients (43.9 vs. 72.2%; log-rank test; *p* = 0.024).

## Discussion

In this study, we have evaluated the importance of the codon 72 polymorphism in p53 on clinical outcome in cancer of the vagina and vulva as well as its association with HPV status, obtained from previous studies. We found that the significance of the polymorphism differed between the two cohorts. Despite a higher primary cure rate for tumors with the arg/arg genotype, distant recurrences were significantly more common among patients where tumors had the arg/arg genotype compared with the arg/pro genotype in the vaginal carcinoma group. For patients with the arg/arg genotype, in the vulvar carcinomas, tumors were significantly larger at diagnosis compared to the arg/pro genotype. In both cohorts, the arg/arg genotype numerically indicated a worse cancer-specific survival, but a statistically significant association was found in the vulvar HPV-negative group only.

Codon 72 polymorphism of p53 did not have a significant association with HPV status in our study. A nonsignificant higher frequency of arginine homozygotes compared to heterozygotes was noted in the HPV-positive vaginal carcinoma patients. To further elude potential impact of p53 polymorphism in relation to HPV status, survival rate was analyzed in both patient groups. Interestingly, for patients with vulvar carcinoma, positive for HPV, the allelic variant arg/arg had no impact on survival. Potentially, HPV is the major prognostic factor for these patients, and not the investigated polymorphism. On the other hand, for patients with HPV-negative vulvar tumors and the arg/arg polymorphism, we show a significantly worse cancer-specific survival rate. Despite the significant finding in univariate analysis, increased tumor size in arg/arg patients was not an independent factor in multivariate analysis (data not shown) and has therefore probably not affected the survival outcome. Few studies have addressed the prognostic value of this polymorphism with studies focusing on HPV-negative cases being almost absent. However, a worse overall survival and lower progression-free survival have been observed in a cohort of nasopharyngeal carcinoma with the arg/arg genotype [28].

To further analyze an association between the codon 72 polymorphism and HPV, different HPV genotypes were compared, with no significant differences. Duin et al. [29] performed a study on cervical carcinoma patients that showed increased risk for developing cancer for women homozygous for arginine with the 350T variant of HPV-16. Unfortunately, our earlier performed HPV-16 sub-genotyping yielded too small groups to identify any association and therefore no statistical analysis could be performed to verify or challenge the previous results. The impact of HPV and p53 codon 72 polymorphism on patient prognosis remains an intriguing subject for further studies.

We previously showed that the tumors expressing mixed histology in vulvar squamous cell carcinomas had a significant worse survival than the other histological types and was associated with an unfavorable tumor stage distribution with less stage I and more stage IV tumors than the other histological types [11]. In the present study, we showed an association of arginine homozygosity with the mixed type of histology, higher tumor stage, a significantly larger tumor size at diagnosis and a numerically worse survival outcome. A higher tumor stage and larger size at diagnosis have earlier been shown to be significant prognostic markers for survival [8–11, 14], and the examined polymorphism may be an additional factor in combination with these clinical tumor parameters.

Sullivan et al. [25] showed that patients with head and neck cancer carrying the arginine allele had a better response to chemo-radiotherapy than patients with other alleles. This is in agreement with our results in the vaginal cohort where higher primary cure rate was seen in arginine homozygotes. Further support for this result is the in vitro studies investigating the impact of the polymorphism of codon 72 on p53 function finding that the p53arg induces apoptosis to a greater extent and with a faster kinetics than p53pro [21, 25, 30, 31]. When exposed to stress, p53arg is transported out of the nucleus and localizes to the mitochondria, while p53pro to a higher extent remains in the nucleus [30, 32, 33]. Deletion of the proline-rich region has been shown to strongly impede the apoptotic function of the p53 protein [34], resulting in a stronger affinity of p53 to MDM2 with higher susceptibility to ubiquitination almost solely occurring in the nucleus [35]. The mitochondrial localization gives a position advantage for p53arg to interact with pro-apoptotic proteins residing at the mitochondrial surface, while the nucleus-located p53pro might experience higher MDM2 degradation [30, 32, 33].

In contrast to Sullivan et al., our superior primary cure rate in vaginal cancer patients, homozygous for arginine, was not associated with increased survival and these patients also had a higher recurrence rate. In comparison with the cohort used by Sullivan et al., where all patients were treated with chemo-radiotherapy, almost all vaginal cancer patients in the current study were treated with radiation alone. Also, 72% of the vulvar cancer patients were treated with primary surgery and adjuvant radiotherapy. Statistical analysis showed that the different treatments did not affect either recurrence rate or survival outcome in the vaginal carcinoma patients (data not shown). An interesting result in our study was the pronounced significance of the arginine genotype in the distant recurrences of vaginal carcinomas. This leads to the assumption that the arginine homozygous tumor cells may have a biological advantage for spreading compared with the other groups. Different expression levels between the polymorphic variants as an explanation are unlikely since metastases have been shown to have lower p53 expression than their primary tumor [36] and levels between the arginine and proline variant have been shown to be equal [21, 30]. A more plausible explanation is the observed difference in protein interactions between arginine and proline. Differences in interactions inducing transcriptional activity of certain apoptotic target genes have been observed, with the p53arg being more potent [31, 33]. On the contrary, p53pro has a higher affinity to p53 inhibitors and is more susceptible to MDM2-mediated degradation, but is also more efficient at inducing cell cycle arrest and DNA repair than p53arg [21, 25, 30].

The most recognized function of p53 is the induction of apoptosis, but it also plays a role in a wide variety of cellular processes, of which many are yet to be fully understood [37]. It has been shown that p53 is more effective at inducing a wider variety of proteins when present at several locations [38]. Since the intracellular locations and structures of arginine and proline variants have been shown to differ, their protein interactions and affinity might also vary.

p53 has also been implicated in the control of other proteins involved in cell motility and invasiveness [39, 40]. p53 can affect cell motility through affecting the PI3-kinase/Rac1 pathway in the presence of mitogenic factors in both normal cells and tumor cells [41] and forms a complex with MDM2 and an invasion promotor named SLUG, an E-cadherin transcription inhibitor, leading to degradation of the complex [42]. As previously mentioned, p53 homozygous for arginine at codon 72 appears to be resistant to MDM2-mediated degradation compared to the proline variant. If this resistance is due to alterations in the binding of MDM2 to p53, then the same scenario might be present in the complex containing SLUG.

A possible explanation for diverging results between the vaginal and vulvar tumor types might be a tissue-specific response in the vagina and vulva, reflecting the use of different pathways of apoptosis in different tissues although both being squamous cell carcinomas. The effect on apoptosis, visualized in mouse models, has shown that p53arg is superior in skin and intestine, p53pro more prominent in the thymus and with no difference in the spleen [33, 43]. p53 has also shown various induction of proteins in different tissues [44], indicating that a tissue-specific predisposition for several pathways used by p53 might be present.

Some limitations of our study are worth to acknowledge. Due to the rareness of the investigated cancers, the cohorts used are small and the inflated false positive or negative rates needs to be considered. When using formalin-fixed and paraffin-embedded tissue, there is also a risk of DNA fragmentation [45]. The unclassifiable results might be caused by loss of p53 wildtype or loss of heterozygosity. Reports have shown a preferential retention of the arginine allele compared to the proline allele [46]. The prevalence of these potential limitations was not further evaluated in this study.

In conclusion, we report different results between vaginal and vulvar carcinomas regarding the p53 codon 72 polymorphism. For vaginal cancer, recurrence rate is affected while survival is left unperturbed in the complete series, and also in HPV-positive and HPV-negative cases when analyzed separately. On the contrary, the survival outcome is affected in HPV-negative vulvar cancer, but not in HPV-positive cases. Larger tumor size was also seen in arginine homozygous patients. This study is to our knowledge the first investigating this relationship, and more comprehensive studies are needed to further analyze the importance of p53 codon 72 polymorphism as a clinical prognostic factor in established carcinomas of the vagina and vulva.

## References

[1] Mutter GL, Prat J. Pathology of the female reproductive tract. 2014. p. 79–159

[2] Wakeham K, Kavanagh K (2014). The burden of HPV-associated anogenital cancers. Gynecol Cancers.

[3] Smith JS, Backes DM, Hoots BE (2009). Human papillomavirus type-distribution in vulvar and vaginal cancers and their associated precursors. Obs Gynecol.

[4] Alonso I, Felix A, Torné A, Fusté V, Castillo P, Balasch J (2012). Human papillomavirus as a favorable prognostic biomarker in squamous cell carcinomas of the vagina. Gynecol Oncol.

[5] Alonso I, Fusté V, Castillo P, Torné A, Fusté P, Rios J (2011). Does human papillomavirus infection imply a different prognosis in vulvar squamous cell carcinoma?. Gynecol Oncol.

[6] Ruttkay-Nedecky B, Jimenez Jimenez AM, Nejdl L, Chudobova D, Gumulec J, Masarik M (2013). Relevance of infection with human papillomavirus: the role of the p53 tumor suppressor protein and E6/E7 zinc finger proteins (Review). Int J Oncol.

[7] Walboomers JMM, Jacobs MV, Manos MM, Bosch FX, Kummer JA, Shah KV (1999). Human papillomavirus is a necessary cause of invasive cervical cancer worldwide. J Pathol.

[8] Hellman K, Lindquist D, Ranhem C, Wilander E, Andersson S (2014). Human papillomavirus, p16INK4A, and Ki-67 in relation to clinicopathological variables and survival in primary carcinoma of the vagina. Br J Cancer.

[9] Brunner AH, Grimm ÞC, Polterauer S, Hefler L, Stani J, Heinze G (2011). The prognostic role of human papillomavirus in patients with vaginal cancer. Int J Gynecol Cancer.

[10] Larsson GL, Helenius G, Andersson S, Sorbe B, Karlsson MG (2013). Prognostic impact of human papilloma virus (HPV) genotyping and HPV-16 subtyping in vaginal carcinoma. Gynecol Oncol.

[11] Larsson GL, Helenius G, Andersson S, Elgh F, Sorbe B, Karlsson MG (2012). Human papillomavirus (HPV) and HPV 16–variant distribution in vulvar squamous cell carcinoma in Sweden. Int J Gynecol Cancer.

[12] Škamperle M, Kocjan BJ, Maver PJ, Seme K, Poljak M (2013). Human papillomavirus (HPV) prevalence and HPV type distribution in cervical, vulvar, and anal cancers in Central and Eastern Europe. Acta Dermatovenerol Alp Pannon Adriat.

[13] Herfs M, Yamamoto Y, Laury A, Wang X, Nucci MR, Mclaughlin-drubin ME (2012). A discrete population of squamocolumnar junction cells implicated in the pathogenesis of cervical cancer. Proc Natl Acad Sci U S A.

[14] Phillips JL, Menck HR, Carolina S (1998). The national cancer data base report on cancer of the vagina. Am Coll Surg Comm Cancer Am Cancer Soc.

[15] Yugawa T, Kiyono T (2009). Molecular mechanisms of cervical carcinogenesis by high-risk human pappilomaviruses: novel functions of E6 and E7 oncoproteins. Rev Med Virol.

[16] Hanahan D, Weinberg RA (2011). Hallmarks of cancer: the next generation. Cell.

[17] Whibley C, Pharoah PD, Hollstein M (2009). p53 polymorphisms: cancer implications. Nat Rev Cancer.

[18] Shi H, Tan S, Zhong H, Hu W, Levine A, Xiao C (2009). Winter temperature and UV are tightly linked to genetic changes in the p53 tumor suppressor pathway in Eastern Asia. Am J Hum Genet.

[19] Andersson S, Rylander E, Strand A, Sällström J, Wilander E (2001). The significance of p53 codon 72 polymorphism for the development of cervical adenocarcinomas. Br J Cancer.

[20] Storey A, Thomas M, Kalita A, Harwood C, Gardiol D, Mantovani F (1998). Role of a p53 polymorphism in the development of human papilloma- virus-associated cancer. Nature.

[21] Ozeki C, Sawai Y, Shibata T, Kohno T, Okamoto K, Yokota J (2011). Cancer susceptibility polymorphism of p53 at codon 72 affects phosphorylation and degradation of p53 protein. J Biol Chem.

[22] Jee SH, Won SY, Yun JE, Lee JE, Park JS, Ji SS (2004). Polymorphism p53 codon-72 and invasive cervical cancer: a meta-analysis. Int J Gynecol Obstet.

[23] Malisic E, Jankovic R, Brotto K, Radulovic S (2013). TP53 codon 72 polymorphism and risk of cervical carcinoma in Serbian women. Arch Gynecol Obstet.

[24] Rosenthal AN, Ryan A, Hopster D, Jacobs IJ (2000). p53 codon 72 polymorphism in vulval cancer and vulval intraepithelial neoplasia. Br J Cancer.

[25] Sullivan A, Syed N, Gasco M, Bergamaschi D, Trigiante G, Attard M (2004). Polymorphism in wild-type p53 modulates response to chemotherapy in vitro and in vivo. Oncogene.

[26] XIV World Congress of Gynecology and Obstetrics (1994). (FIGO). Montreal, Canada. Abstracts. Int J Gynaecol Obstet.

[27] Tavassoli FA, Devilee P. Pathology and genetics of tumors of the breast and female genital organs. Vol. IARCPress, World Health Organization Classification of Tumors. 2003.

[28] Li ML, Dong Y, Hao YZ, Xu N, Ning FL, Chen SS (2014). Association between p53 codon 72 polymorphisms and clinical outcome of nasopharyngeal carcinoma. Genet Mol Res.

[29] van Duin M, Snijders PJ, Vossen MT, Klaassen E, Voorhorst F, Verheijen RH (2000). Analysis of human papillomavirus type 16 E6 variants in relation to p53 codon 72 polymorphism genotypes in cervical carcinogenesis. J Gen Virol.

[30] Dumont P, Leu JI, Della AC, Iii P, George DL, Murphy M (2003). The codon 72 polymorphic variants of p53 have markedly different apoptotic potential. Nat Genet.

[31] Thomas M, Kalita ANN, Labrecque S, Pim D, Banks L, Matlashewski G (1999). Two polymorphic variants of wild-type p53 differ biochemically and biologically. Mol Cell Biol.

[32] Altilia S, Santoro A, Malagoli D, Lanzarini C, Ballesteros JA, Galazzo G (2012). TP53 codon 72 polymorphism affects accumulation of mtDNA damage in human cells. Aging (Albany NY).

[33] Zhu F, Dollé MET, Berton TR, Kuiper RV, Capps C, Espejo A (2011). Mouse models for the p53 R72P polymorphism mimic human phenotypes. Cancer Res.

[34] Sakamuro D, Sabbatini P, White E, Prendergast GC (1997). The polyproline region of p53 is required to activate apoptosis but not growth arrest. Oncogene.

[35] Berger M, Sionov RV, Levine AJ, Haupt Y (2001). A role for the polyproline domain of p53 in its regulation by Mdm2. J Biol Chem.

[36] Pickhard A, Gröber S, Haug AK, Piontek G, Wirth M, Straßen U (2014). Survivin and pAkt as potential prognostic markers in squamous cell carcinoma of the head and neck. Oral Surg Oral Med Oral Pathol Oral Radiol.

[37] Lee C-L, Blum JM, Kirsch DG (2013). Role of p53 in regulating tissue response to radiation by mechanisms independent of apoptosis. Transl Cancer Res.

[38] Pu T, Zhang XP, Liu F, Wang W (2010). Coordination of the nuclear and cytoplasmic activities of p53 in response to dna damage. Biophys J.

[39] Chen H, Yuan Y, Zhang C, Luo A, Ding F, Ma J (2012). Involvement of S100A14 protein in cell invasion by affecting expression and function of matrix metalloproteinase (MMP)-2 via p53-dependent transcriptional regulation. J Biol Chem.

[40] Hwang C-I, Matoso A, Corney DC, Flesken-Nikitin A, Körner S, Wang W (2011). Wild-type p53 controls cell motility and invasion by dual regulation of MET expression. Proc Natl Acad Sci U S A.

[41] Sablina AA, Chumakov PM, Kopnin BP (2003). Tumor suppressor p53 and its homologue p73α affect cell migration. J Biol Chem.

[42] Wang S-P, Wang W-L, Chang Y-L, Wu C-T, Chao Y-C, Kao S-H (2009). p53 controls cancer cell invasion by inducing the MDM2-mediated degradation of slug. Nat Cell Biol.

[43] Azzam GA, Frank AK, Hollstein M, Murphy ME (2011). Tissue-specific apoptotic effects of the p53 codon 72 polymorphism in a mouse model. Cell Cycle.

[44] Fei P, Bernhard EJ, El-Deiry WS (2002). Tissue-specific induction of p53 targets in vivo. Cancer Res.

[45] Williams C, Pontén F, Moberg C, Söderkvist P, Uhlén M, Pontén J (1999). A high frequency of sequence alterations is due to formalin fixation of archival specimens. Am J Pathol.

[46] Brooks LA, Tidy JA, Gusterson B, Hiller L, O’Nions J, Gasco M (2000). Preferential retention of codon 72 arginine p53 in squamous cell carcinomas of the vulva occurs in cancers positive and negative for human papillomavirus. Cancer Res.

